# Current knowledge of SLC6A1-related neurodevelopmental disorders

**DOI:** 10.1093/braincomms/fcaa170

**Published:** 2020-10-13

**Authors:** Kimberly Goodspeed, Eduardo Pérez-Palma, Sumaiya Iqbal, Dominique Cooper, Annalisa Scimemi, Katrine M Johannesen, Arthur Stefanski, Scott Demarest, Katherine L Helbig, Jingqiong Kang, Frances C Shaffo, Brandon Prentice, Catherine A Brownstein, Byungchan Lim, Ingo Helbig, Emily De Los Reyes, Dianalee McKnight, Vincenzo Crunelli, Arthur J Campbell, Rikke S Møller, Amber Freed, Dennis Lal

**Affiliations:** f1 Children's Health, Medical Center, UT Southwestern, Dallas, TX 75235, USA; f2 Department of Pediatrics, Medical Center, UT Southwestern, Dallas, TX 75235, USA; f3 Genomic Medicine Institute, Lerner Research Institute Cleveland Clinic, Cleveland, OH 44195, USA; f4 Broad Institute of MIT and Harvard, Stanley Center for Psychiatric Research, Cambridge, MA 02142, USA; f5 Broad Institute of MIT and Harvard, Center for Development of Therapeutics, Cambridge, MA 02142, USA; f6 Department of Biological Sciences, University at Albany, Albany, NY 12222, USA; f7 Department of Epilepsy Genetics and Personalized Treatment, Danish Epilepsy Center Filadelfia, Dianalund 4293, Denmark; f8 Department of Regional Health Research, Institute for Regional Health Services, University of Southern Denmark, Odense, Denmark; f9 Departments of Pediatrics and Neurology, University of Colorado School of Medicine, Aurora, CO, USA; f10 Department of Pediatric Neurology and Neuroscience Institute, Children's Hospital Colorado, Aurora, CO, USA; f11 Division of Neurology, Children’s Hospital of Philadelphia, Philadelphia, PA 19104, USA; f12 The Epilepsy NeuroGenetics Initiative (ENGIN), Children's Hospital of Philadelphia, Philadelphia, PA, USA; f13 Department of Biomedical and Health Informatics (DBHi), Children’s Hospital of Philadelphia, Philadelphia, PA 19104, USA; f14 Department of Neurology, Vanderbilt University Medical Center, TN 37232, USA; f15 SLC6A1 Connect Foundation, Denver, CO 80210, USA; f16 Division of Genetics and Genomics, Department of Pediatrics, Boston Children's Hospital and Harvard Medical School, Boston, MA, USA; f17 Department of Pediatrics, Seoul National University College of Medicine, Seoul National University Hospital, Seoul 03080, Republic of Korea; f18 Department of Neurology, Perelman School of Medicine, University of Pennsylvania, Philadelphia, PA, 19104, USA; f19 Department of Pediatric Neurology, Nationwide Children's Hospital, Columbus, OH, USA; f20 The Ohio State University College of Medicine, Columbus, OH, USA; f21 GeneDx, Gaithersburg, MD 20877, USA; f22 Neuroscience Division, School of Bioscience, Cardiff University, Cardiff, UK; f23 Faculty of Medicine and Surgery, Malta University, Msida, Malta; f24 Neurological Institute, Epilepsy Center, Cleveland Clinic, Cleveland, OH 44195, USA

**Keywords:** *SLC6A1* haploinsufficiency, *SLC6A1*-related disorders, GABA transporter 1, neurodevelopmental disorders, seizures

## Abstract

Advances in gene discovery have identified genetic variants in the solute carrier family 6 member 1 gene as a monogenic cause of neurodevelopmental disorders, including epilepsy with myoclonic atonic seizures, autism spectrum disorder and intellectual disability. The solute carrier family 6 member 1 gene encodes for the GABA transporter protein type 1, which is responsible for the reuptake of the neurotransmitter GABA, the primary inhibitory neurotransmitter in the central nervous system, from the extracellular space. GABAergic inhibition is essential to counterbalance neuronal excitation, and when significantly disrupted, it negatively impacts brain development leading to developmental differences and seizures. Aggregation of patient variants and observed clinical manifestations expand understanding of the genotypic and phenotypic spectrum of this disorder. Here, we assess genetic and phenotypic features in 116 individuals with solute carrier family 6 member 1 variants, the vast majority of which are likely to lead to GABA transporter protein type 1 loss-of-function. The knowledge acquired will guide therapeutic decisions and the development of targeted therapies that selectively enhance transporter function and may improve symptoms. We analysed the longitudinal and cell type-specific expression of solute carrier family 6 member 1 in humans and localization of patient and control missense variants in a novel GABA transporter protein type 1 protein structure model. In this update, we discuss the progress made in understanding and treating solute carrier family 6 member 1-related disorders thus far, through the concerted efforts of clinicians, scientists and family support groups.

## Teaser

Genetic variants in the *SLC6A1* gene have been identified as a monogenic cause of neurodevelopmental disorders, including epilepsy with myoclonic atonic seizures, autism spectrum disorder and intellectual disability. Here, we assess the genetic and phenotypic features observed in 116 patients with *SLC6A1* variants.

## Graphical Abstract

**Graphical Abstract:**
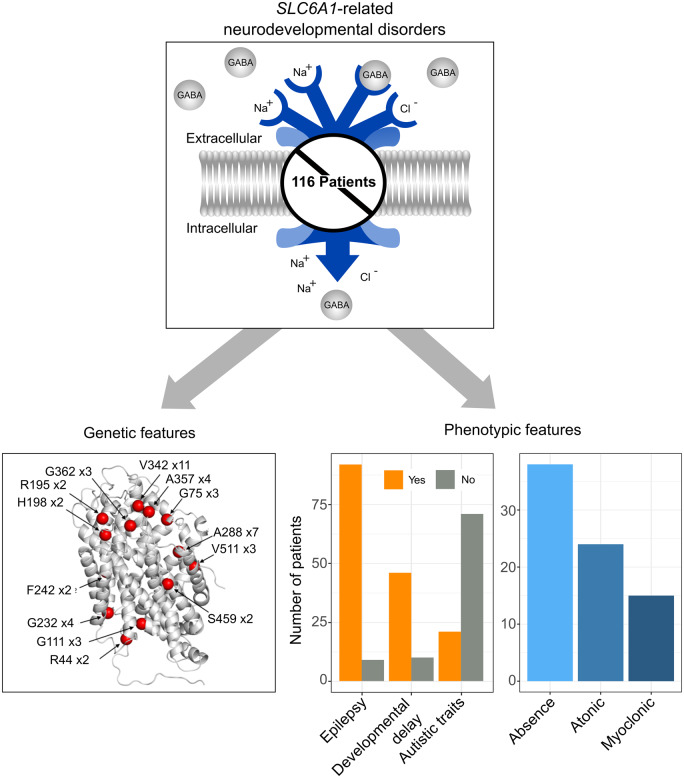

## Introduction

Solute carrier family 6 member 1 (*SLC6A1*)-related disorders are emerging as a common cause of developmental and epileptic encephalopathies, since initial descriptions in 2015 (Carvill *et al.*, 2015). *SLC6A1* encodes the GABA transporter protein type 1 (GAT1), which is responsible for the reuptake of GABA into presynaptic neurons and glia (Bröer and Gether, 2012). Disruption of *SLC6A1* is a prominent cause of neurodevelopmental disorders, including autism spectrum disorder, intellectual disability and seizures of varying types and severity. In the current three largest genomic screens of individuals with epilepsy (8565, 9170 and 9769 patients, respectively), *SLC6A1* was listed among the top 10–20 genes with the highest number of pathogenic variants (Lindy *et al.*, 2018; Epi25 Collaborative, 2019, p. 25; Truty et al., 2019)*.* In the most extensive autism sequencing study to date (*N* = 11 986), *SLC6A1* was among the top 10 genes, with the most significant variant enrichment in autism patients compared to 23 598 controls (Satterstrom *et al.*, 2019). Recently, exome sequencing of individuals with schizophrenia found rare *de novo* missense variants in *SLC6A1* to be associated with schizophrenia in three patients (Rees *et al.*, 2020), extending the phenotype spectrum beyond epilepsy. Overall, the incidence of *SLC6A1*-related disorders is estimated to be 2.65 (90%CI: 2.38–2.86) per 100 000 births(López-Rivera *et al.*, 2020).

Following the second *SLC6A1* Symposium organized by the *SLC6A1* Connect Foundation (https://slc6a1connect.org, 1 October 2020, date last accessed) that convened academic scientists, physicians and family advocacy organizations, we summarize the current state of research and future directions. We collected and curated the largest dataset of *SLC6A1* variants to date with accompanying clinical phenotyping (*N* = 116). Our study represents a significant step forward to define the clinical and genotypic spectrum of *SLC6A1*-related disorders and ultimately will help to guide clinical management.

## The *SLC6A1* gene

**Figure 1:**
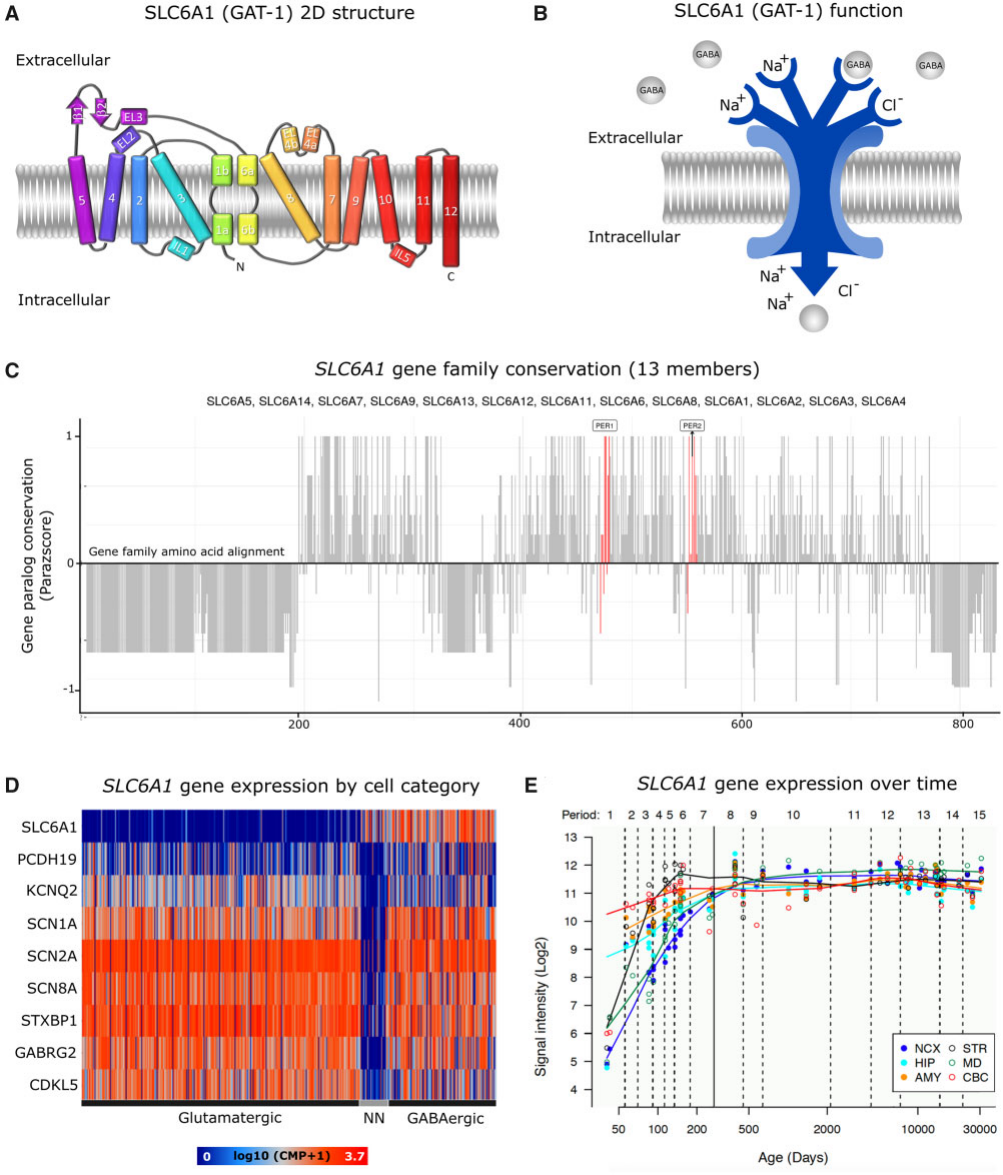
***SLC6A1* main features.** (**A**) Schematic representation of the 2D structure of GAT1. (**B**) Diagram of GAT1 GABA transport function. (**C**) Paralogue conservation of the *SLC6A1* gene family members. Higher values denote the degree of conservation. The pathogenic enriched regions are shown in red bars. (**D**) Cell type-specific expression of *SLC6A1* and other frequently mutated epilepsy and neurodevelopmental disorder-associated genes (http://celltypes.brain-map.org/rnaseq/human). (**E**) Dynamic gene expression of *SLC6A1* along with entire development and adulthood in the cerebellar cortex (CBC), mediodorsal nucleus of the thalamus (MD), striatum (STR), amygdala (AMY), hippocampus (HIP) and 11 areas of the neocortex (NCX) (http://hbatlas.org).

The *SLC6A1* gene is located on chromosome 3 (Genome Research Consortia human assembly version 38 genomic coordinates: 3:10 992 733–11 039 248) and contains 15 exons. The encoded GAT1 protein has 12 transmembrane domains that form a single chain transporter (Fig. 1A). The primary function of GABA transporters is to lower the concentration of GABA in the extracellular space (Scimemi, 2014) (Fig. 1B). There are six major splice isoforms of human GAT1 that differ by alternative use of exons three to five. The transcript ENST00000287766 is the longest isoform of *SLC6A1* and is considered canonical (Hunt *et al.*, 2018); accordingly, most genetic variants are mapped into its sequence. The exact topology of GAT1 remains unclear due to the lack of a mammalian crystal structure. Still, homology models based on a 20–25% sequence identity to GAT1 (Yamashita *et al.*, 2005) have allowed the identification of essential residues for substrate and sodium binding in transmembrane domains one, three, six and eight, which are necessary for the conformational transitions during the transport process (Fig. 1A and B). The *SLC6A1* gene belongs to a gene family of 20 paralogues. The proteins encoded by 13 of these genes exhibit above 80% sequence identity, and six of them can transport GABA with different degrees of substrate specificity. These paralogues have been associated with a variety of neurodevelopmental disorders (Bröer and Gether, 2012). Upon linear protein sequence alignment of the 13 most paralogue-conserved gene family members, disease-associated variants reported in different family members cluster significantly together in two amino acid regions in comparison to variants from the general population. These regions are known as pathogenic variant enriched regions and any missense variant found within is 106 times more likely to be classified as pathogenic than benign (Fig. 1C) (Pérez-Palma *et al.*, 2020). However, as in the case of many other genetic etiologies linked to neurodevelopmental disorders, disease-causing variants in *SLC6A1* among affected individuals are broadly distributed along its sequence (Johannesen *et al.*, 2018).

## The function of GAT-1

The *SLC6A1* gene is expressed in the mammalian central nervous system, predominantly in the adult frontal cortex (Gamazon *et al.*, 2018). Unlike other GABA transporters, GAT1 is almost exclusively expressed in GABAergic axon terminals but can also be present in astrocytes, oligodendrocytes and microglia (Minelli *et al.*, 1995; Melone *et al.*, 2015; Fattorini *et al.*, 2017, 2020) (Fig. 1D). During embryonic development, GAT1 is expressed within fully functional GABAergic neurons even before glutamatergic excitatory activity, but complete development of the GABAergic system continues through adolescence (Kilb, 2012; Wu and Sun, 2015) (Fig. 1E). GABA transporters couple translocation of GABA with the dissipation of the electrochemical gradient for sodium and chloride. By moving these ions across the membrane in a fixed ratio with GABA (1 GABA: 2 Na^+^: 1 Cl^−^), GAT1 generates a stoichiometric current (Lester *et al.*, 1994) (Fig. 1B). At rest, the driving force for sodium and chloride forces these ions to move from the extracellular space towards the cell cytoplasm, promoting rapid binding and intracellular translocation of extracellular GABA within milliseconds of its release. This fast removal system prevents GABA from activating neighboring synapses (Isaacson *et al.*, 1993). In addition to the transport of GABA, GAT1 is also an ion channel that generates two ionic currents: (i) an inward sodium current activated by GABA binding to GAT1 and (ii) a leak current mediated by alkali ions, which occurs in the absence of GABA (Risso *et al.*, 1996; MacAulay *et al.*, 2002). Lastly, in the absence of GABA, GAT1 generates a sodium-dependent capacitive current (Mager *et al.*, 1993). Through these currents, GAT1 activation can create local changes in membrane potential or a shunt for membrane resistance. In GAT1 deficient mice, tonic inhibition is increased and the decay time of evoked phasic currents mediated by GABA_A_ receptors is prolonged, whereas the frequency, amplitude and kinetics of spontaneous GABA_A_ postsynaptic currents is not changed (Jensen *et al.*, 2003; Chiu *et al.*, 2005; Bragina *et al.*, 2008; Cope *et al.*, 2009).

Interestingly, other works on GAT1-deficient mice show that the frequency of miniature inhibitory postsynaptic currents is decreased, an effect that is associated with increased expression of enzymes that contributes to GABA synthesis in presynaptic terminals of inhibitory neurons (i.e. GAD65/67, periaqueductal gray matter) (Bragina *et al.*, 2008; Conti *et al.*, 2011). Other works in the striatum indicate that GAT1 shape GABAergic transmission through pre- and post-synaptic mechanisms. Together, these findings highlight multiple molecular and cellular functions of GAT1 that demonstrate the complex etiology leading to clinical symptoms of patients with *SLC6A1*-related disorders. Given that GABA homeostasis is critical for brain development, it is plausible that early intervention strategies will be essential for the well-being of the patients.

## *SLC6A1*-related disorders

In 2015, a 3p microdeletion involving only *SLC6A1* and *SLC6A11* was described in a patient with Doose Syndrome, a developmental epileptic encephalopathy associated with intellectual disability and early-onset epilepsy with myoclonic atonic seizures (previously myoclonic atonic epilepsy). Furthermore, likely pathogenic variants in *SLC6A1* were identified in 6/160 (4%) individuals with a previously undiagnosed early-onset epilepsy with myoclonic atonic seizures (Carvill *et al.*, 2015) and additional studies identified individual patients with autism spectrum disorder and developmental epileptic encephalopathy carrying variants in *SLC6A1* (Rauch *et al.*, 2012; Sanders *et al.*, 2012). In the first case series, 34 patients with variants in *SLC6A1* were clinically described in 2018 (Johannesen *et al.*, 2018). Notably, nearly all genetic variants reported arose *de novo* and not present in the general population (Lek *et al.*, 2016).

**Figure 2:**
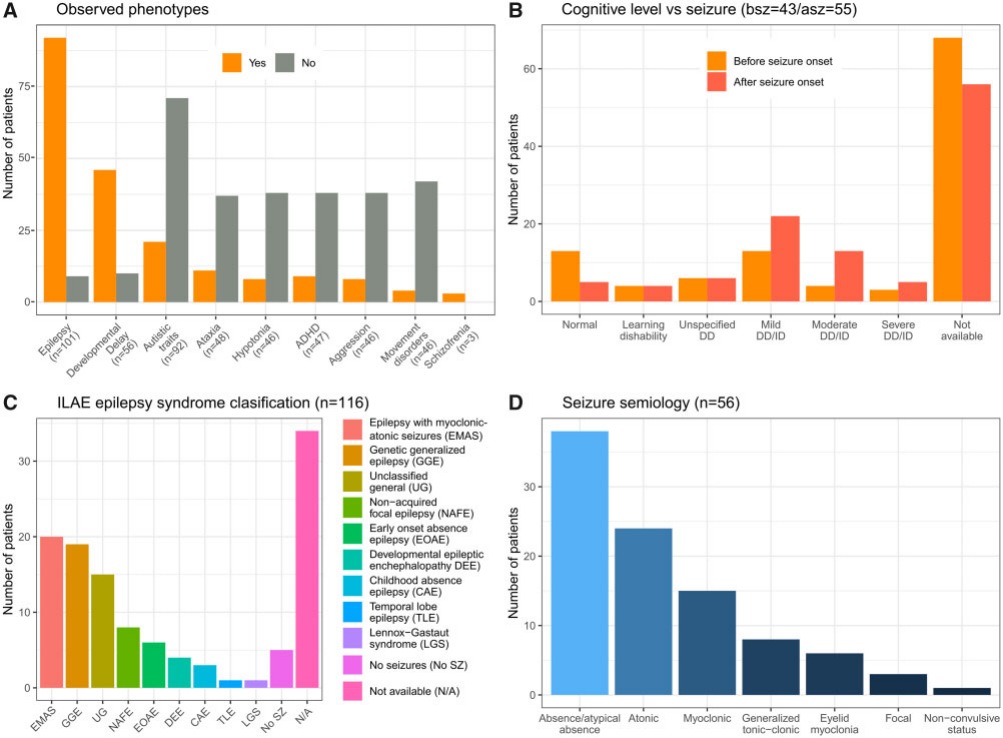
**Updated *SLC6A1* cohort of patient’s summary statistics.** We collected 116 patients from three different sources: the literature, **Epi25 Collaborative for Large-Scale Whole Genome Sequencing in Epilepsy**, and direct contact with the patient family foundation *SLC6A1* connect. A total of 85 unique *SCL6A1* variants were observed, and the clinical phenotype is expanded, and all clinical features are summarized in Supplementary Table 1. When data are available, we show (**A**) the phenotypes observed including additional clinical features beyond epilepsy, (**B**) the developmental status of patients before and after the onset of seizures, (**C**) the distribution of epilepsy classification (per updated International League Against Epilepsy guidelines) and (**D**) reported seizure semiologies.

Here we present the largest collection of *SLC6A1* patients, including genetic and clinical features (Supplementary Table 1). Our cohort includes 116 patients from three sources: previously reported individuals (58.6%), individual referrals to the *SLC6A1* Connect Foundation (15.5%), and the Epi25 Collaborative for Large-Scale Whole Genome Sequencing in Epilepsy Collaborative Database (25.8%) (Epi25 Collaborative, 2019). Clinical and genetic data are presented in Supplementary Table 1. Data from the Epi25 Collaborative for Large-Scale Whole Genome Sequencing in Epilepsy Collaborative database were limited to genotype and International League Against Epilepsy categorization only. We describe 85 unique *SLC6A1* variants. Most of patients variants observed arose *de novo* (40/49, 81.63%). Regarding variant type, we observed 88 missense variants, 15 protein truncating variants) that lead to a complete loss of function, seven variants in splice sites, three large deletions Copy Number Variants, two small insertions and deletions and one synonymous variant (Supplementary Table 1). In our cohort, epilepsy (92/101, 91.1%), developmental delay, and cognitive impairment (46/56, 82.1%) and autistic traits (20/92, 22.8%) were the most common clinical features (Fig. 2A). We found a similar number of males and females (55.1% female), and the mean age of seizure onset was 2.5 years (standard deviation 1.58 years). Developmental data concerning seizure-onset was available on 43 before seizure-onset and 55 after seizure-onset. Developmental delay was present in 26/43 (60.4%) before seizure-onset. After the onset of seizures, nearly all subjects demonstrate developmental delay (46/55, 83.6%), most in the mild to moderate range (35/55, 63.6%) (Fig. 2B). The most prevalent epilepsy syndrome was early-onset epilepsy with myoclonic atonic seizures (20/82, 24.3%) followed by genetic generalized epilepsy (19/82, 23.1%) and non-acquired focal epilepsy (8/82, 9.75%) (Fig. 2C). Detailed seizure semiology data was available on 56 of the 92 subjects with epilepsy; most commonly (atypical) absence seizures (38/53, 71.7%), atonic seizures (24/54, 44.44%) and myoclonic seizures (15/54, 27.77.1%) (Fig. 2D). Generalized epileptiform discharges (36/52, 69.2%) were the most commonly reported EEG abnormality among available data, especially at a frequency of 2–4 Hz (12/52, 23%). Generalized background slowing is reported in 17/52 (32.6%). Recently, an exome-wide trio sequencing study identified *de novo* missense variants in S*LC6A1* to be associated with schizophrenia (Rees *et al.*, 2020). No evidence of epilepsy, intellectual disability, or autism spectrum disorders was reported in the three patients described (Supplementary Table 1).

## Towards a model of *SLC6A1* pathophysiology

Missense variants can cause a gain of function or loss of function; however, the expected molecular mechanism by which variants lead to *SLC6A1*-related disorders is loss of function or haploinsufficiency. This disease-model is supported by *in vivo* and *in vitro* experiments in both wild type and GAT1^−/−^ mice, as well as studies on recombinant GAT1 proteins from individuals with *SLC6A1* variants. However, the mechanisms by which the loss of function lead to clinical manifestations are not well understood. Recently, experimental evidence showed that seven *SLC6A1* variants (five missense variants, one in-frame deletion and one nonsense variant) identified in epilepsy patients reduce GABA transport *in vitro* studies (Mattison *et al.*, 2018). The residual transporter activity was found, ranging from 2% to 27% compared to the wild type.

It has been shown that *SLC6A1* variants also cause impaired protein trafficking. Characterization of a missense variant in *SLC6A1* (G234S) associated with Lennox–Gastaut syndrome leads to reduced protein expression in both cell surface and total protein levels in heterologous and rat cortical neurons (Cai *et al.*, 2019). The surface protein level of the mutant is about 70% of the wild type, while the GABA uptake of the mutant GAT1 is ∼30% of the wild type. This suggests the mutant GAT1 had impaired GABA uptake in addition to impaired protein trafficking (Cai *et al.*, 2019). Similarly, a recent report on *SLC6A1* (P361T) associated with epilepsy and autism indicates that the mutant GAT-1 had endoplasmic reticulum retention and enhanced degradation (Wang et al., 2020).

There is no specific animal model of *SLC6A1*-related disorders. Heterozygous (Het) GAT1 knockout mice are phenotypically normal despite having diminished GABA reuptake capacity (Chiu *et al.*, 2005; Cope *et al.*, 2009). The homozygous GAT1 knockout animals exhibit a constant tremor, abnormal gait, reduced strength, absence seizures and mobility, as well as anxious behaviors (Chiu *et al.*, 2005; Cope *et al.*, 2009). This model partially recapitulates features of the human disease, including mobility and cognitive impairment (Johannesen *et al.*, 2018). *Ex vivo* hippocampal and thalamic recordings show that GAT1 homozygous knockout mice have an increase in GABA_A_R tonic inhibition (Jensen *et al.*, 2003; Cope *et al.*, 2009), which is known to contribute to generalized spike-wave discharge typical of absence seizure(Crunelli V *et al.*, 2020).

## Prediction of GAT1 structure and evaluation of missense variants

**Figure 3:**
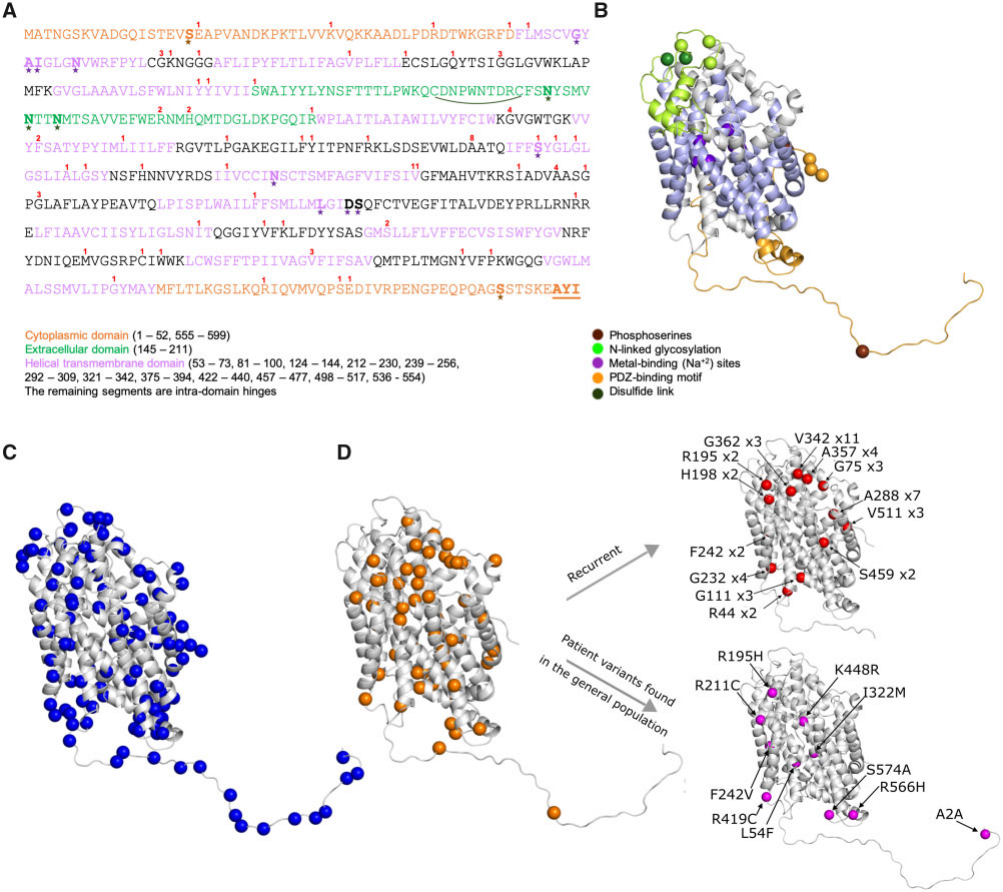
**GAT1 3D structure model and spatial distribution of patient and general population missense variants in *SLC6A1*.** There is no experimentally solved protein structure of the eukaryotic GAT1 protein (Fig. 2A). We predicted the GAT-1 structure using a homology model from a dopamine transporter (Protein Data Bank ID: 4xp4) (Waterhouse et al., 2018) and a multi-template based serotonin transporter (Protein Data Bank IDs: 4xpt, and 6awn) using RaptorX web server (Wang et al., 2016). The resulting novel model for GAT1 exhibited high quality and confidence (*P*-value = 3.67e−13). (**A**) Linear GAT1 protein sequence. The 599-residue long sodium- and chloride-dependent GAT1 consists of 12 helical domains (light purple), 1 extracellular (light green) and 2 cytoplasmic (light orange). (**B**) Predicted 3D structure, domains (colored as in **A**), and special binging sites (colored spheres). Numbers (in red) above the reference amino acids in sequence reflect the count of patients carrying a missense variant altering that amino acid. (**C)** Genome aggregation consortia variants (blue spheres) variant positions mapped on 3D and (**D)** patient variants (orange spheres). Recurrent variants are highlighted in red (upper-left structure) as well as patient variants also found in the general population (*N* = 10, bottom-left structure).

Truncating variants are very likely to lead to loss-of-function due to lower levels of GAT1 and subsequent haploinsufficiency. However, the molecular consequence of missense variants can range from benign to damaging, mostly determined by the effect of the variants on protein function. We explored the pathogenic variants’ positions in the GAT1 sequence in the context of the functionally critical sites and domains (Fig. 3A). To further investigate the benign variants from the general population and pathogenic variants from patients in 3D space, we generated the structure of GAT1 using the RaptorX server [Fig. 3B, based on template structure: 4XPT (Wang et al., 2015)]. We then mapped the variants’ positions on the structure (Fig. 3C and D). Differential spatial segregation of variants is notable upon 3D mapping. The majority of the patient variants are located in the helical-transmembrane segments (32 out of 88, 36.4%) and inter-helical hinges (42 out of 88, 47.8%) (Fig. 3D, left) while genome aggregation consortia variants cluster in the cytoplasmic domain (38 out of 121, 31.4%, Fig. 3C). We observed ten patient variants also present in the general population (genome aggregation consortia) (Supplementary Table 1). The pathogenicity of these variants can be challenged according to the ACMG guidelines (Richards *et al.*, 2015). We further highlighted the amino acid positions harboring recurrent patient variants and those also found in genome aggregation consortia (Fig. 3D, right). Out of 13 recurrent variant positions, six positions form a cluster near the extracellular (top) part of the structure. All three schizophrenia-related variants’ positions are located on the outer surface of the structure, mutated residues more than 70% exposed to solvent as measured by the residue’s accessible surface area.

## Existing treatment

Available clinical data for individuals with disease-causing variants in *SLC6A1* is limited, and most patients require an interdisciplinary team, including neurologists, developmental pediatricians, genetic counsellors and speech and occupational therapists for comprehensive management (Kuo *et al.*, 2016; Katkin *et al.*, 2017). There is insufficient data available to guide pharmacotherapy in *SLC6A1*-related disorders. Thus treatment is guided by existing strategies for the specific clinical epilepsy syndromes, rather than underlying genetic etiology, using broad-spectrum anti-seizure medications, including valproic acid, lamotrigine or benzodiazepines. In a prior study, 20 of 31 patients became seizure-free with anti-seizure medication, and valproic acid was the most effective drug. Lamotrigine and ethosuximide also showed success (Johannesen *et al.*, 2018). Importantly, there was not a correlation between seizure control and cognitive outcome.

## Future treatment

There is a clear unmet medical need for improved treatment options for *SLC6A1*-related disorder. GAT1 is a potential candidate for therapeutic development based on the following observations: (i) it has a known biological function based on *in vitro* and *in vivo* studies (Jensen *et al.*, 2003; Scimemi, 2014), (ii) there are known antiepileptic drugs such as valproic acid, tiagabine and vigabatrin that can modulate GABA concentrations (Schousboe *et al.*, 2014) and (iii) the transporters’ relatively small size (599 aa) makes *SLC6A1* a plausible candidate for viral-mediated gene therapy using adeno-associated virus vectors (Chamberlain *et al.*, 2016). Alternatively, antisense oligonucleotides therapy can be used to specifically increase productive *SLC6A1* mRNA and consequently restore levels of GAT1 protein. Antisense oligonucleotides strategies targeting non-productive splicing events have shown promising results in animal models of other haploinsufficiency neurodevelopmental disorders like Dravet syndrome (Han *et al*., 2020; Lim *et al.*, 2020).

Restoration of the GAT1 transporter function may provide therapeutic benefit, but two challenges arise. First, is the need to understand the regulation of GAT1 expression and activity in a developmental context, and second, the reversibility of symptoms is unknown. Patient-derived induced pluripotent stem cell experiments (i.e. *in vitro* neuronal cultures and organoids) allow researchers to probe for the functional effects of *SLC6A1* variants on neuronal excitability, developmental progression and network behavior. Importantly, this can be done in human neurons, under physiological expression levels of the transporter and under each patient’s unique genetic background. Transgenic rodent models provide the opportunity to assay neurons in the context of the brain to capture non-autonomous, circuit-level and behavioral effects. Understanding how alterations in GAT1 function affect both developing and mature neuronal integration, as well as network function, is critical to enable effective interventions.

## Towards precision therapeutics

To develop treatments for patients with *SLC6A1*-related disorders it is critical to define the full phenotypic spectrum of the disease. Observational studies are needed to characterize the natural course of the disease and to identify appropriate end-points for use in future interventional trials. This cross-sectional review of available clinical data is a critical first step. Based on the expanded cohort described here, epilepsy and neurodevelopmental disabilities appear to be the core features of *SLC6A1*-related disorders. However, additional assessments of gait and mobility or movement disorders may also be of interest.

Further progress will require collaboration between clinicians, scientists and family organizations such as the *SLC6A1*-Connect Foundation (https://slc6a1connect.org). Significant limitations of a retrospective dataset include the lack of uniformity of presented data and risk that the same patient may be presented in multiple de-identified datasets. Collaborations between crucial stakeholders will lead to the development of disease-specific tools for collecting and analysing patient data, including greater data consistency, longitudinal phenotyping and standardized evaluation of medication use and response. More extensive population studies may also elucidate relationships between *SLC6A1* variants and milder phenotypes. Furthermore, the use of EEG and imaging biomarkers can be explored. EEG may prove to be a valuable biomarker, given the high prevalence of abnormal electrical activity in clinical reports, however little is known about the EEG signature in individuals with *SLC6A1*-related disorder without seizures. Finally, standardized neuropsychological assessments to characterize developmental disabilities are needed, including patient-centred outcome measures that are jointly developed with substantial input from patient advocates and family organizations.

A key challenge in the pursuit of precision therapeutics is the identification of eligible individuals. While access to genetic testing is improving, it remains a significant barrier in some countries. Identification of early phenotypic features that should prompt appropriate evaluation will support early diagnosis. Alongside deep phenotyping, functional analysis of variants will be necessary to assess genotype-phenotype correlations. Finally, the implementation of high-throughput analysis of the functional impact of variants on channel function would accelerate drug development considerably.

## Conclusion

*SLC6A1*-related disorders are neurodevelopmental disorders caused by aberrant GABA neurotransmission secondary to impaired functioning of GAT1. Based on a review of 116 individuals with *SLC6A1*-related disorder, developmental delay, epilepsy, autism and motor dysfunction, including stereotypies and ataxia, are the most common clinical features. Data from the literature and our analysis on GAT1 structure support loss-of-function as the primary disease-associated molecular pathology. A comprehensive translational research programme needs to be developed (i) to better understand the underlying pathophysiology, (ii) to develop targeted therapies for *SLC6A1*-related disorder and (iii) to define the full clinical spectrum of the disease.

## Supplementary material

Supplementary material is available at *Brain Communications* online.

## Supplementary Material

fcaa170_Supplementary_Data

## Abbreviations

GAT1 =: GABA transporter protein type 1
SLC6A1 =: solute carrier family 6 member 1
